# Violet light could improve sleep quality and blood glucose levels in healthy individuals: A pilot study

**DOI:** 10.1371/journal.pone.0314346

**Published:** 2025-08-21

**Authors:** Tomonobu Kato, Yasue Mitsukura

**Affiliations:** Faculty of Science and Technology of Keio University, Yokohama, Kanagawa, Japan; University of Nebraska Medical Center College of Medicine, UNITED STATES OF AMERICA

## Abstract

Light is perceived through the retina, the light-sensing tissue layer of the eye, and can directly influence the brain. Light exposure triggers various biological responses that improve sleep quality and stabilize mood. Recently, violet light, exhibiting a unique wavelength of 360–400 nm, has garnered significant attention due to its perception through a specific receptor, marking a novel research area. Herein, we used “violet light glasses” that directly emit violet light into the human eye and investigated their effects on healthy humans in real-life settings. This study demonstrated that two hours of violet light exposure in the morning enhanced sleep quality in approximately 20% of the healthy participants and was also associated with improved blood glucose levels. Although this was a pilot study due to its small sample size, our findings indicate that violet light could potentially promote health benefits in humans.

## Introduction

Artificial light effects on humans have been debated [1], using both pro and con arguments. Light can positively affect the circadian clock [2], is effective for sleep problem treatment [3], and can also affect mood, light therapy is thus widely used for mood disorder treatment [4]. However, inappropriate light exposure at inappropriate times, such as that of LED screens and smartphones, can interfere with sleep [5,6]. In addition, short-wavelength (e.g., ultraviolet; i.e., UVA: 320–400 nm, UVB: 280–320 nm, and UVC: 100–280 nm [7] as well as blue, i.e., 380–500 nm [8]) light reportedly exert toxic effects.

Recently, violet (360–400 nm) light has gained attention for its myopia-suppressing effects and has been proven safe for humans [9]. Recent evidence has revealed that it affects not only the eyes but the entire brain. In mice, it reportedly affects the brain positively, e.g., improves memory and depressive-like behaviors [10] through *OPN5*-positive retinal ganglion cells [11], by affecting the nucleus accumbens and paraventricular thalamic nucleus via the habenular region. In another case, *OPN5*-positive hypothalamic preoptic neurons sense violet light and suppress heat production in the brown adipose tissue [12]. In mammals, including humans, *OPN5* is expressed in the retina and brain [13], comprising deep brain regions such as the hypothalamus [14]. Therefore, violet light expectably affects the entire brain via the OPN5 receptor in the human retina and deep brain regions. However, how violet light affects humans have not been sufficiently investigated.

Recently, violet light-emitting glasses have been developed to suppress myopia, providing a method for exposing humans to violet light in a real-life settings (further described in the Materials and Methods section). Based on the above-mentioned considerations, we evaluated how violet light could affect healthy individuals using such glasses. Two hours of bright light exposure in the morning reportedmy alleviates sleep disorders [15]. Moreover, numerous studies have confirmed an association between sleep quality, various diseases, and physiological indicators [16]. For instance, non-rapid eye movement (NREM) sleep quality is associated with glucose homeostasis [17], and insomnia is a risk factor for type 2 diabetes [18]. Moreover, as the hypothalamus, i.e., an *OPN-5*-positive area, is involved in sleep-wake cycle [19] and feeding behavior [20] regulation, violet light itself might retain the potential to affect sleep quality and blood glucose levels. Therefore, in this study, we investigated the sleep quality enhancement potential of violet light and its effect on blood glucose levels through two hours of violet light exposure in the morning. Our results suggest that violet light exposure might potentially improve sleep quality and blood glucose levels.

## Materials and methods

### Data acquisition

This study was approved by the Bioethics Committee of the Faculty of Science and Technology at Keio University (approval ID: 2023−058). The employees of the Social Welfare Corporation (“Smiling Park”) of Japan were recruited. Twenty-five healthy participants (six male and 19 female) volunteered to be included in this study. The recruitment period lasted from April 17, 2023, to May 1, 2023. Only participants with sufficient data were included in the analysis. Data from certain participants were excluded (see below). All healthy participants were Japanese (age: 23–65 years; mean age ± standard deviation [SD]: 42.0 ± 11.6 years). Written informed consent was obtained from all participants. The experiment was conducted continuously in homes and workplaces.

### Materials

#### Heart rate and sleep track sensor.

We used Fitbit Charge 5^©^ (Fitbit Inc., San Francisco, CA, USA), a commercially available wireless fitness-tracking wristband, to record the heart rate and estimate sleep time and sleep stages using tri-axial accelerometry and photoplethysmography.

#### Glucose sensor.

We used Freestyle libre 2^®^ (Abbott Diabetes Care, Alameda, CA, USA), an interstitial fluid glucose flash monitoring system that records blood glucose levels per minute, allowing for stable and continuous blood glucose level measurements for 14 days as a commonly used self-monitoring device in patients with diabetes [21].

#### Tear volume test device.

To determine the tear volume, we used the Strip Meniscometry Tube (SMTube, Echo Electricity, Fukushima, Japan), allowing for noninvasively quantifying the tear volume in the human tear meniscus.

#### Violet light glasses.

We used the TLG-001J glasses (Tsubota Laboratory Incorporated, JINS Inc., Tokyo, Japan), which emits violet light (wavelength: 360–400 nm) into the human eye.

### Experimental protocol

For 10 weeks from the start of the experiment, the participants wore Fitbit Charge 5^©^ all days, and from weeks three to sixth, violet light glasses for two hours in the morning. Moreover, the participants put the Freestyle libre 2^®^ on their right or left upper arm from the first day to the second week, from the fourth week to the sixth week, and from the eighth week to the tenth week. Tear volume was measured at the end of the second, sixth, and tenth weeks.

### Data processing

We only included data from participants who met the following criteria: (1) data available for at least three days within a two-week period and (2) data available for at least 80% of each day. Outliers were defined as values that deviated from the median by more than three times the median absolute deviation (MAD), as this method is robust for extreme values [22].

### Statistical analysis

All experimental data were analyzed using the following parametric statistics: two-sample *t*-test, one-way analysis of variance (ANOVA) followed by the Tukey–Kramer *post-hoc* test, repeated measures ANOVA followed by the Tukey–Kramer *post-hoc* test, and a linear mixed-effects model.

We used the MATLAB software (MathWorks, version R2023a) for data analysis, processing, and statistics.

### Sleep quality assessment

#### Difficulty in falling asleep.

We defined difficulty in falling asleep as sleep onset latency, corresponding to the time required for falling asleep [23].

#### Nocturnal awakening.

One of the indicators used to assess sleep quality is the ‘degree of fragmentation’ [24]. In this study, we defined the ‘degree of fragmentation’ as the number of nocturnal awakenings during the night.

#### Early morning awakening.

We defined early morning awakening as temporary waking up within 2 h of the usual hour of waking, based on the GRID-HAMD-17 criteria [25,26]

## Results

### Violet light improves self-satisfaction with sleep experience

#### Verbal questionnaire.

First, we examined whether violet light improved sleep quality. The latter is commonly characterized by the self-satisfaction of an individual with their sleep experience, displaying four objective indices: sleep efficiency, latency, and duration as well as wake after sleep onset [24]. In this study, we examined the self-satisfaction of the participants concerning their sleep and investigated the objective indices of individuals who experienced sleep quality improvement. Objective indices were measured using Fitbit, yielding over 60% accuracy [27], typically used as a simplified tool for sleep research.

**Fig 1:**
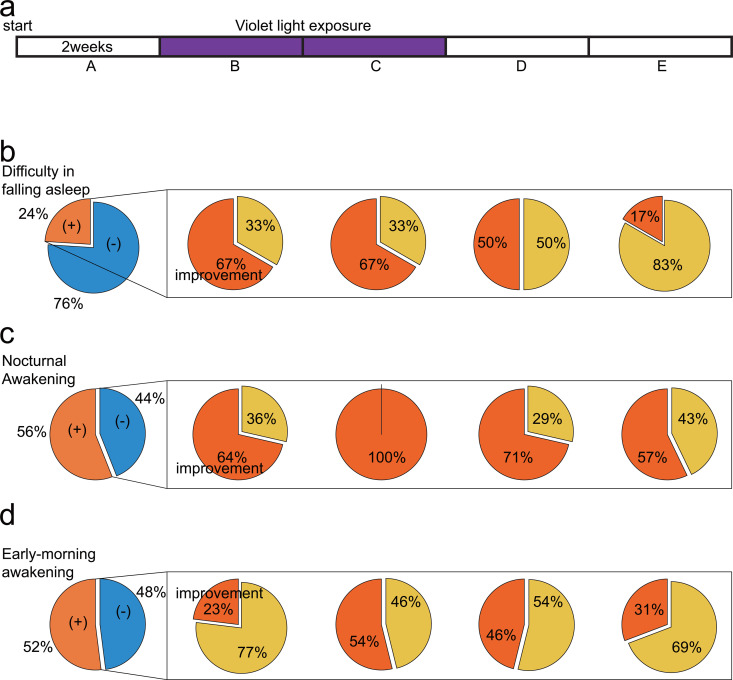
Time course of improvement in sleep satisfaction along with violet light exposure. **(a)** Time course of the experiment. The left column presents sleep problem rates in the control period, i.e., (b) difficulty in falling asleep, (c) nocturnal awakening, and (d) early morning awakening. The right column highlights corresponding improvements during and after violet light exposure.

In this study, we initially collected biometric data (using the Fitbit and Freestyle Libre) for two weeks. During this period, no interventions were conducted, and each participant maintained their normal routines. Therefore, we used such data as a control. Next, each participant was exposed to violet light for 2 h in the morning for one month, then no interventions were conducted in the upcoming month. Using this protocol, we aimed to determine biometric information-related alterations before and after the intervention and assess the duration of such alterations upon the intervention. We named the assessed time periods as A (i.e., control period), B (from the start of violet light exposure for 2 weeks), C (from weeks 3–4 after the start of violet light exposure), D (from the end of violet light exposure until week 2 post-exposure), and E (from week 3–4 post-exposure) (Fig 1a), referring to them as such in the figures.

Using a verbal questionnaire, more than half of the participants experienced sleep problems, such as difficulty falling asleep, nocturnal awakenings, and early morning awakenings (Fig 1b–1d).

Among the 25 participants, six experienced trouble falling asleep. After violet light exposure, 67% of them experienced an improvement in their symptoms, with the effects diminishing by approximately 50% two weeks after violet light exposure and nearly disappearing four weeks post-exposure (Fig 1b). Fourteen out of the 25 participants suffered from nocturnal awakenings; 36% of them experienced an improvement in their symptoms two weeks after light exposure. All participants experienced improvement in nocturnal awakenings four weeks after light exposure. The effects gradually regressed after the cessation of light exposure, yet sustained approximately 60% of the effects four weeks post-exposure (Fig 1c). Thirteen of the 25 participants experienced early morning awakening. After two weeks of exposure, 23% experienced improvement, increasing to 54% after four weeks. Even after two weeks post-light exposure, approximately half of the participants still felt sustained effects, and approximately 30% felt continued improvement four weeks post-exposure (Fig 1d).

Next, we analyzed biometric data, focusing on 19 participants for whom data was available for all sleep indicators for more than 80% of the entire experimental period.

#### Difficulty in falling asleep.

**Fig 2:**
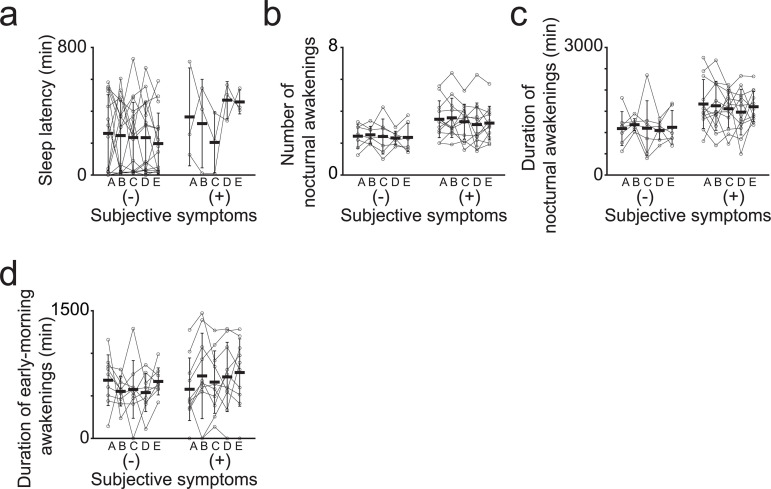
Improvement in each sleep quality index upon violet light exposure. **(a)** Time course of sleep latency of each participant. Tables 2 and 3 present statistical test results. **(b)** Number of nocturnal awakenings in each participant. Tables 6 and 8 present statistical test results. **(c)** Duration of nocturnal awakenings in each participant. Tables 7 and 9 present statistical test results. **(d)** Duration of early morning awakenings in each participant. Tables 11 and 12 present statistical test results. A, B, C, D, and E correspond to the time period presented in Fig 1. Error bars indicate the standard deviation.

Among the 19 participants, three displayed troubles falling asleep; we thus categorized them as the symptomatic group and the others as the non-symptomatic group. We excluded outliers (defined as elements more than three scaled MAD from the median; the same applies hereafter) and revealed that the average sleep latency was 364.5 ± 306.8 h in the symptomatic group and 261.0 ± 242.6 h in the non-symptomatic group (mean ± SD; Fig 2a, Table 1, time period A). After violet light exposure, sleep latency decreased to 322.7 ± 277.6 and 247.6 ± 218.6 h (mean ± SD; symptomatic and non-symptomatic group, respectively; Fig 2a, Table 1, time period B) within two weeks after the start of light exposure. Next, from two to four weeks after exposure, sleep latency further decreased to 204.5 ± 190.9 and 235.1 ± 221.0 h (mean ± SD; symptomatic and non-symptomatic group, respectively; Fig 2a, Table 1, time period C). Subsequently, two weeks after and from two to four weeks after the end of exposure, sleep latencies in the symptomatic group changed to 469.6 ± 115.8 and 458.2 ± 75.1 h, while those in the non-symptomatic group to 234.4 ± 224.3 and 197.5 ± 191.2 h, respectively (mean ± SD; Fig 2a, Table 1, time periods D and E).

**Table 1:** Sleep latency tme course in each participant.

		Sleep latency (min)				
Symptoms	Subject number	Time period				
		A	B	C	D	E
(+)	4	127.5 ± 146.8	11.3 ± 15.5	8.6 ± 14.6	503.1 ± 436.5	424.3 ± 455.1
	14	255.0 ± 176.2	544.3 ± 356.5	390.0 ± 232.0	340.7 ± 228.4	544.3 ± 592.4
	23	711.0 ± 346.4	412.5 ± 243.8	215.0 ± 232.3	565.0 ± 599.0	406.2 ± 318.8
(-)	2	22.5 ± 15.0	605.0 ± 501.6	12.9 ± 16.0	30.0 ± 19.0	30.0 ± 0
	5	0	16.7 ± 15.8	20.0 ± 24.5	0	30.0 ± 46.9
	8	18.0 ± 16.4	11.3 ± 15.5	22.5 ± 21.2	30.0 ± 0	302.1 ± 292.9
	13	40.0 ± 55.9	373.8 ± 246.4	493.8 ± 440.3	555.0 ± 368.9	180.0 ± 206.4
	15	430.0 ± 298.0	486.7 ± 411.6	225.0 ± 172.3	335.5 ± 281.7	290.0 ± 224.5
	16	525.0 ± 477.8	18.8 ± 15.5	19.1 ± 15.1	33.3 ± 46.1	48.8 ± 80.1
	17	350.0 ± 291.4	10.0 ± 15.5	90.0 ± 114.2	18.8 ± 15.5	17.1 ± 16.0
	18	240.0 ± 268.8	530.8 ± 384.1	258.0 ± 356.4	671.5 ± 508.3	460.9 ± 295.5
	19	545.0 ± 306.2	466.2 ± 400.9	399.2 ± 246.9	450.0 ± 457.7	220.9 ± 216.4
	20	311.3 ± 327.8	30.0 ± 0	346.2 ± 385.5	15.0 ± 16.0	18.0 ± 16.4
	21	0	64.3 ± 80.2	25.0 ± 12.2	82.5 ± 108.5	20.0 ± 15.0
	24	540 ± 405.6	302.5 ± 273.6	368.6 ± 243.6	458.6 ± 298.5	493.8 ± 244.5
	25	552.9 ± 495.0	366.0 ± 368.5	728.6 ± 676.3	375.0 ± 291.4	590.0 ± 480.2
	26	582.0 ± 123.0	366.9 ± 327.3	252.0 ± 135.8	317.5 ± 274.5	290.8 ± 215.8
	27	0	13.3 ± 21.8	15.0 ± 15.8	73.3 ± 114.3	145.0 ± 157.1
	29	20.0 ± 17.3	300.0 ± 188.7	486.4 ± 396.1	330.0 ± 176.0	22.5 ± 13.9

**Table 2:** Sleep latency-related statistical analysis results in each time period for the group analysis.

		Symptoms(+)	Symptoms(-)
Comparison		*p* Value	
A	B	1	1
	C	0.89	0.99
	D	0.94	1
	E	0.98	0.78
B	C	0.48	1
	D	0.93	1
	E	0.85	0.88
C	D	0.60	1
	E	0.26	0.94
D	E	1	0.89

*p < 0.05.

**Table 3:** Sleep latency-related statistical analysis results in each time period for each participant.

		*p* Value																		
		Symptoms (+)			Symptoms(-)															
Comparison		Subject number																		
		4	14	23	2	5	8	13	15	16	17	18	19	20	21	24	25	26	27	29
A	B	0.96	0.32	0.34	0.02*	0.77	1	0.22	1	2.2 × 10^−5^*	4.1 × 10^−4^*	0.45	0.99	0.19	0.44	0.45	0.93	0.47	1	0.47
	C	0.96	0.91	0.02*	1	0.70	1	0.04*	0.83	9.5 × 10^−6^*	4.5 × 10^−3^*	1	0.90	1	0.97	0.72	0.96	0.12	1	4.7 × 10^−2^*
	D	0.11	0.98	0.89	1	1	1	0.02*	0.97	2.6 × 10^−5^*	3.2 × 10^−4^*	0.11	0.98	0.17	0.19	0.98	0.98	0.27	0.67	0.35
	E	0.29	0.32	0.31	1	0.22	0.03*	0.91	0.90	5.9 × 10^−5^*	3.9 × 10^−4^*	0.73	0.33	0.29	0.98	1	1	0.18	0.06	1
B	C	1	0.83	0.69	2.0 × 10^−3^*	1	1	0.87	0.60	1	0.56	0.45	0.99	0.06	0.80	0.98	0.56	0.80	1	0.41
	D	0.02*	0.58	0.85	4.7 × 10^−3^*	0.71	1	0.62	0.80	1	1	0.88	1	1	0.98	0.63	1	0.99	0.69	1
	E	0.06	1	1	2.8 × 10^−3^*	0.82	6.8 × 10^−3^*	0.60	0.64	1	1	0.99	0.39	1	0.65	0.45	0.90	0.93	0.03*	0.17
C	D	0.02*	1	0.16	1	0.65	1	0.99	0.97	1	0.59	0.09	1	0.05	0.47	0.92	0.77	0.97	0.69	0.56
	E	0.08	0.83	0.70	1	0.95	0.01*	0.15	1	1	0.60	0.75	0.69	0.13	1	0.78	0.99	1	0.03*	1.6 × 10^−3^*
D	E	0.97	0.58	0.82	1	0.16	4.5 × 10^−2^*	0.06	1	1	1	0.67	0.55	1	0.29	1	0.96	1	0.47	0.09

*p < 0.05.

**Table 4:** Nocturnal awakening number in each participant.

		Number of nocturnal awakenings				
Symptoms	Subject number	Time period				
		A	B	C	D	E
(+)	2	3.35 ± 1.10	2.58 ± 1.44	2.50 ± 1.31	2.44 ± 1.42	3.38 ± 1.12
	4	3.26 ± 1.06	2.91 ± 1.70	4.17 ± 2.12	1.50 ± 0.71	2.64 ± 1.28
	5	2.78 ± 0.97	3.56 ± 1.33	1.57 ± 0.79	2.20 ± 1.32	2.00 ± 0
	8	5.57 ± 3.05	6.40 ± 2.01	5.29 ± 0.95	6.29 ± 0.95	5.71 ± 2.43
	13	2.00 ± 0	2.77 ± 1.54	2.00 ± 0	2.60 ± 1.26	3.00 ± 1.41
	14	3.50 ± 1.65	3.00 ± 0	3.58 ± 1.24	3.64 ± 1.39	2.33 ± 0.78
	18	4.22 ± 1.39	5.07 ± 1.44	4.33 ± 1.83	4.23 ± 1.69	3.92 ± 1.44
	19	3.33 ± 1.51	2.92 ± 1.12	3.31 ± 1.80	3.22 ± 1.72	4.14 ± 0.69
	25	5.40 ± 1.14	4.33 ± 1.50	3.71 ± 2.50	3.50 ± 1.73	3.80 ± 0.84
	26	4.00 ± 1.41	4.08 ± 1.85	4.00 ± 0	2.40 ± 0.97	3.07 ± 1.49
	27	3.00 ± 0.63	3.50 ± 1.38	3.55 ± 1.21	4.33 ± 1.23	3.14 ± 1.41
	29	2.00 ± 0	1.92 ± 1.44	2.14 ± 1.17	1.62 ± 0.87	1.92 ± 1.04
(-)	15	3.33 ± 1.63	2.88 ± 0.83	4.25 ± 1.26	3.00 ± 0	3.75 ± 1.39
	16	3.00 ± 0.63	2.82 ± 1.17	2.92 ± 1.16	2.58 ± 1.16	3.33 ± 1.50
	17	3.00 ± 0.63	3.40 ± 2.50	3.00 ± 0.94	2.55 ± 0.82	2.27 ± 1.49
	20	1.71 ± 0.95	2.36 ± 1.50	1.85 ± 1.46	1.92 ± 0.95	1.63 ± 1.19
	21	1.25 ± 0.71	2.00 ± 1.41	1.00 ± 0	1.78 ± 0.97	1.77 ± 1.36
	23	2.56 ± 1.01	2.29 ± 1.38	2.45 ± 1.04	2.18 ± 1.78	2.33 ± 1.37
	24	2.20 ± 1.10	1.92 ± 1.38	1.45 ± 0.93	2.29 ± 1.38	1.43 ± 1.02

**Table 5:** Nocturnal awakening duration in each participant.

		Duration of nocturnal awakenings (min)				
Symptoms	Subject number	Time period				
		A	B	C	D	E
(+)	2	1558.9 ± 451.3	972.5 ± 592.9	1075.0 ± 548.4	1103.3 ± 773.5	1812.3 ± 1009.5
	4	1606.3 ± 356.1	1150.9 ± 875.0	1942.5 ± 1022.0	495.0 ± 210.4	1418.6 ± 1091.4
	5	1320.0 ± 608.2	1430.0 ± 397.7	732.9 ± 416.7	1263.0 ± 899.7	1384.3 ± 1211.6
	8	2442.9 ± 1196.1	2697.0 ± 1074.1	2031.4 ± 499.5	2331.4 ± 471.1	2318.6 ± 1163.1
	13	798.0 ± 128.3	1479.2 ± 873.6	1488.8 ± 1231.3	1194.0 ± 563.5	1323.0 ± 605.2
	14	1560.0 ± 804.6	1410.0 ± 325.9	1795.0 ± 607.4	1742.1 ± 761.9	1177.5 ± 425.8
	18	2330.0 ± 1040.7	2136.4 ± 753.7	1960.0 ± 986.8	1873.8 ± 667.1	2000.0 ± 716.4
	19	1395.0 ± 736.5	1490.8 ± 634.7	1350.0 ± 859.5	1826.7 ± 1116.9	1555.7 ± 112.8
	25	2760.0 ± 544.2	2163.3 ± 478.9	1662.9 ± 1144.3	1635.0 ± 706.8	1680.0 ± 784.3
	26	1775.0 ± 777.8	2164.6 ± 938.4	2120.0 ± 597.5	1321.0 ± 586.2	1988.6 ± 874.0
	27	1455.0 ± 101.7	1745.0 ± 568.2	1590.0 ± 542.5	2112.5 ± 519.5	1380.0 ± 655.2
	29	1070.0 ± 426.7	697.5 ± 506.6	957.9 ± 465.7	846.9 ± 589.8	1236.9 ± 747.0
(-)	15	1815.0 ± 1079.0	1117.5 ± 310.4	2347.5 ± 1166.9	1332.9 ± 214.1	1653.8 ± 829.7
	16	1230.0 ± 555.6	1254.5 ± 889.0	1277.5 ± 725.1	1207.5 ± 671.8	1684.2 ± 674.1
	17	1155.0 ± 63.6	1443.0 ± 1095.0	1194.0 ± 509.7	1020.0 ± 303.6	1107.3 ± 776.5
	20	557.1 ± 332.2	1011.4 ± 628.2	858.5 ± 803.1	826.2 ± 544.5	870.0 ± 583.5
	21	761.3 ± 636.0	1077.3 ± 823.8	395.0 ± 189.2	693.3 ± 383.7	867.7 ± 642.1
	23	1140.0 ± 678.0	1172.1 ± 944.8	1123.6 ± 640.0	1145.5 ± 1001.2	987.5 ± 626.6
	24	990.0 ± 648.0	1210.0 ± 1168.0	510.0 ± 324.8	1116.4 ± 980.7	687.9 ± 595.5

**Table 6:** Statistical analysis results of the nocturnal awakening numbers in each time period in group analysis.

		Symptoms(+)	Symptoms(-)
Comparison		*p* Value	
A	B	0.99	0.99
	C	0.93	1
	D	0.80	0.94
	E	0.85	0.99
B	C	0.86	1
	D	0.42	0.70
	E	0.62	0.97
C	D	0.97	1
	E	1	1
D	E	1	1

*p < 0.05.

**Table 7:** Statistical analysis results of the nocturnal awakening durations in each time period in the group analysis.

		Symptoms(+)	Symptoms(-)
Comparison		*p* Value	
A	B	1	0.96
	C	0.93	1
	D	0.78	0.99
	E	0.98	1
B	C	0.98	1
	D	0.72	0.54
	E	1	0.99
C	D	0.99	1
	E	0.99	1
D	E	0.90	0.96

*p < 0.05

**Table 8:** Statistical analysis results of the nocturnal awakening numbers in each participant in each time period.

Comparison		*p* Value																		
		Symptoms (+)												Symptoms(-)						
		Subject number																		
		2	4	5	8	13	14	18	19	25	26	27	29	15	16	17	20	21	23	24
A	B	0.59	0.98	0.53	0.93	0.75	0.90	0.72	0.98	0.79	1	0.92	1	0.94	1	1	0.81	0.61	0.99	0.99
	C	0.49	0.56	0.18	1	1	1	1	1	0.46	1	0.91	1	0.73	1	1	1	0.99	1	0.78
	D	0.50	0.05	0.76	0.97	0.90	1	1	1	0.47	0.20	0.23	0.98	0.98	0.98	1	1	87	0.97	1
	E	1	0.83	0.59	1	0.57	0.17	0.99	0.85	0.58	0.67	1	1	0.96	0.99	0.97	1	0.84	1	0.73
B	C	1	0.26	5.4 × 10^−3^*	0.82	0.63	0.82	0.76	0.96	0.95	1	1	0.99	0.31	1.00	0.98	0.83	0.42	1	0.89
	D	1	0.20	0.06	1	1	0.75	0.64	0.90	0.92	0.05	0.50	0.96	1	0.99	0.71	0.90	0.99	1	0.93
	E	0.52	1	0.04*	0.93	0.99	0.74	0.35	0.40	0.98	0.36	0.95	1	0.55	0.85	0.46	0.69	0.99	1	0.84
C	D	1	8.1 × 10^−4^*	0.75	0.90	0.84	1	1	1	1	0.20	0.57	0.74	0.42	0.96	0.96	1	0.69	0.99	0.43
	E	0.42	0.08	0.94	0.99	0.43	0.09	0.97	0.74	1	0.67	0.93	0.99	0.95	0.92	0.82	1	0.64	1	1
D	E	0.44	0.34	1	0.98	0.95	0.06	0.99	0.72	1	0.78	0.13	0.96	0.71	0.57	0.99	0.98	1	1	0.33

*p < 0.05.

**Table 9:** Statistical analysis results of the nocturnal awakening durations in each time period in each participant.

Comparison		*p* Value																		
		Symptoms (+)												Symptoms(-)						
		Subject number																		
		2	4	5	8	13	14	18	19	25	26	27	29	15	16	17	20	21	23	24
A	B	0.26	0.68	1	0.98	0.49	0.99	0.98	1	0.64	0.86	0.83	0.86	0.44	1	0.99	0.52	0.80	1	0.99
	C	0.46	0.85	0.55	0.94	0.55	0.91	0.85	1	0.14	0.94	0.99	1	0.80	1	1	0.84	0.80	1	0.82
	D	0.59	0.02*	1	1	0.89	0.96	0.71	0.83	0.22	0.81	0.13	0.97	0.77	1	1	0.89	1	1	1
	E	0.90	0.98	1	1	0.75	0.62	0.89	1	0.21	0.98	1	0.99	0.99	0.87	1	0.87	1	1	0.96
B	C	1	0.16	0.38	0.66	1	0.67	0.98	0.99	0.70	1	0.96	0.79	0.08	1	0.94	0.97	0.21	1	0.27
	D	0.99	0.37	0.99	0.94	0.91	0.76	0.92	0.86	0.78	0.11	0.48	0.97	0.98	1	0.66	0.94	0.64	1	1
	E	0.03*	0.92	1	0.89	0.99	0.93	0.99	1.00	0.79	0.98	0.45	16	0.61	0.63	0.82	0.99	0.92	0.98	0.50
C	D	1	1.2 × 10^−3^*	0.62	0.98	0.93	1	1	0.63	1	0.32	0.17	0.98	0.22	1	0.98	1	0.89	1	0.37
	E	0.08	0.49	0.50	0.97	0.99	0.13	1	0.98	1	1	0.88	0.72	0.56	0.66	1	1	0.53	0.99	0.98
D	E	0.16	0.06	1	1	1	0.17	1	0.96	1	0.28	0.01*	0.44	0.92	0.51	1	1	0.97	0.99	0.65

*p < 0.05.

Next, we conducted a group analysis, i.e., comparison between the symptomatic and non-symptomatic groups. However, we observed no significant differences (Table 2; repeated measures ANOVA, followed by the Tukey–Kramer *post-hoc* test), possibly due to high-level individual variations (Table 1). However, statistical analysis of individual data (Table 3; one-way ANOVA, followed by the Tukey–Kramer *post-hoc* test) indicated that two participants displayed significantly reduced sleep latency during and after violet light exposure, categorized as the non-symptomatic group. The observed effect in these participants was maintained for at least one month post-exposure (participants 16 and 17). In addition, four participants tended to display reduced sleep latency upon light exposure, although no or only partially significant differences could be observed (participants 4, 23, 24, and 26). Among them, two participants were categorized in the symptomatic group, in which the effect of violet light was attenuated within two weeks after the end of light exposure (participants 13 and 29). In contrast, two participants exhibited significantly increased sleep latency upon light exposure. In summary, these data suggest that violet light exposure could potentially reduce sleep latency in approximately 30% of the healthy individuals, regardless of their symptoms, while only approximately 10% might experience a counterproductive effect.

#### Nocturnal awakening.

Next, we evaluated the frequency and duration of nocturnal awakening. Among all participants, twelve experienced nocturnal awakenings. Hence, we categorized them as the symptomatic group and the others as the non-symptomatic group. After excluding the outliers, the average frequency and duration of nocturnal awakenings were 3.50 ± 1.15 times and 1669.5 ± 575.8 min, respectively, in the symptomatic group, and 2.44 ± 0.76 times and 1092.6 ± 398.7 min, respectively, in the non-symptomatic group (mean ± SD; Figs 2b and 2c, Tables 4 and 5).

We then conducted a group analysis. However, we could observe no significant differences (Tables 6 and 7; repeated measures and one-way ANOVA, followed by the Tukey–Kramer *post-hoc* test), possibly due to large individual variations (Tables 4 and 5). Moreover, our statistical analysis for the individual data (Tables 8 and 9; one-way ANOVA, followed by the Tukey–Kramer *post-hoc* test) generally revealed no significant differences. However, three participants in the symptomatic group tended to have a decreased number of nocturnal awakenings (participants 2, 5, and 25). Moreover, five participants in the symptomatic group and one participant in the non-symptomatic group tended to display reduced nocturnal awakening durations (participants 2, 5, 18, 21, 25, and 29).

Our results (Tables 4–9) do not fully explain why the nocturnal awakenings decreased in almost all symptomatic participants (Fig 1c). Even when limited to the comparison between the control and light exposure at 2–4 weeks later (time period A vs. C), nocturnal awakening durations presented a decreasing trend in nine participants (participants 2, 5, 8, 18, 21, 23, 24, 25, and 29), among whom two exhibited significant differences (paticipants 2 and 5, *p* = 4.7 × 10^−2^ and 0.03, respectively, two-sample *t*-test). To summarize the above-described data, a violet light exposure-related decreasing trend could be generally observed in the nocturnal awakenings approximately 30–50% of the time (6–9 per 19 participants), with no adverse effects observed.

#### Early morning awakening.

Next, we investigated the data regarding early morning awakening. We defined early morning awakening as temporary waking up within 2 h of the usual hour of waking, based on the GRID-HAMD-17 criteria [25,26]. According to these criteria, 10 of 19 participants reported symptoms of early morning awakening during verbal assessment (Fig 1d). After we excluded the outliers, the duration of wakefulness during the early morning was 684.0 ± 297.5 min (mean ± SD; same as below) in the non-symptomatic group before violet light exposure, 553.3 ± 172.4 min from the start of exposure to 2 weeks later, 575.4 ± 339.0 min from 2 weeks after the start of exposure to 4 weeks later; after the end of exposure, 538.6 ± 222.4 min until the two weeks after the end of exposure, and 669.6 ± 158.6 min from 2 weeks to 4 weeks later (Fig 2d and Table 10). These results indicate that violet light might potentially improve early morning awakening, and this effect tended to persist for approximately two weeks after the end of light exposure. However, among the symptomatic group, no consistent trend was observed in the respective time periods (577.3 ± 368.1, 734.5 ± 502.2, 661.5 ± 366.7, 722.3 ± 406.7, 774.4 ± 395.9 min for time periods A, B, C, D, and E, respectively; Fig 2d and Table 10). Furthermore, no statistically significant differences were observed in either case (Table 11; repeated measures ANOVA followed by the Tukey–Kramer *post-hoc* test).

**Table 10:** Duration of early morning awakening in each participant.

Symptoms	Subject number	Duration of early-morning awakenings (min)				
		Time period				
		A	B	C	D	E
(+)	4	774.4 ± 395.9	0	136.7 ± 210.6	0	0
	14	440.0 ± 366.8	548.6 ± 555.6	495.0 ± 311.8	752.1 ± 462.6	597.9 ± 681.6
	16	360.0 ± 262.7	676.4 ± 782.7	385.0 ± 390.1	807.7 ± 575.8	975.0 ± 500.9
	17	345.0 ± 21.2	603.0 ± 488.1	636.0 ± 490.1	597.5 ± 182.5	515.0 ± 258.4
	18	423.8 ± 411.2	0	255.0 ± 274.5	544.6 ± 576.0	410.0 ± 416.2
	21	680.0 ± 707.3	930.0 ± 612.2	1098.8 ± 435.9	1263.3 ± 446.1	1283.3 ± 580.7
	24	1275.0 ± 543.6	1472.3 ± 1091.5	915.0 ± 672.2	1073.6 ± 650.5	807.9 ± 799.6
	25	767.1 ± 835.9	1074.0 ± 899.5	844.3 ± 766.7	562.5 ± 466.9	1062.0 ± 393.2
	26	1020.0 ± 221.8	1390.9 ± 577.3	1269.0 ± 878.6	1287.5 ± 645.5	1202.3 ± 901.4
	29	462.0 ± 363.5	650.0 ± 453.2	580.7 ± 541.4	334.6 ± 285.6	890.8 ± 698.0
(-)	2	661.5 ± 366.7	237.0 ± 325.4	470.0 ± 376.9	548.6 ± 227.0	623.1 ± 444.7
	5	753.0 ± 520.5	580.9 ± 497.9	0	403.3 ± 444.0	656.3 ± 631.6
	8	844.3 ± 1060.0	810.0 ± 554.8	507.0 ± 378.0	773.3 ± 520.9	638.6 ± 531.3
	13	505.7 ± 651.4	461.5 ± 540.2	579.2 ± 450.2	110.0 ± 177.5	423.8 ± 214.8
	15	440.0 ± 427.5	406.7 ± 486.3	405.0 ± 308.4	474.5 ± 475.6	990.0 ± 827.6
	19	815.0 ± 841.6	573.8 ± 417.7	755.5 ± 221.6	863.3 ± 797.9	739.1 ± 546.8
	20	603.8 ± 601.6	527.1 ± 487.5	572.3 ± 437.6	597.7 ± 399.4	588.0 ± 309.3
	23	891.4 ± 250.8	660.0 ± 299.6	600.0 ± 269.4	425.5 ± 251.3	576.9 ± 366.4
	27	142.5 ± 169.5	722.5 ± 719.5	1290.0 ± 901.6	650.8 ± 494.7	790.7 ± 683.4

**Table 11:** Statistical analysis results of the duration of early morning awakenings in each time period in the group analysis.

		Symptoms(+)	Symptoms(-)
Comparison		*p* Value	
A	B	0.28	0.84
	C	0.76	0.97
	D	0.49	0.72
	E	0.36	1
B	C	0.87	1
	D	1	1
	E	0.99	0.60
C	D	0.92	1
	E	0.56	0.92
D	E	0.97	0.40

*p < 0.05.

**Table 12:** Statistical analysis results of the duration of early morning awakenings in each time period in each participant.

Comparison		*p* Value																		
		Symptoms (+)										Symptoms(-)								
		Subject number																		
		4	14	16	17	18	21	24	25	26	29	2	5	8	13	15	19	20	23	27
A	B	6.2 × 10^−8^*	0.99	0.87	0.90	0.24	0.93	0.99	0.92	0.91	0.96	0.09	0.93	1	1	1	0.94	1	0.57	0.27
	C	6.1 × 10^−9^*	1	1	0.86	0.89	1	0.93	1	0.98	0.99	0.74	0.06	0.79	1	1	1	1	0.38	0.01*
	D	9.4 × 10^−7^*	0.63	0.63	0.90	0.96	0.28	0.99	0.99	0.97	0.99	0.97	0.54	1	0.41	1	1	1	0.02*	0.39
	E	2.5 × 10^−8^*	0.95	0.33	0.98	1	0.25	0.84	0.96	0.99	0.50	1	0.99	0.95	1	0.36	1	1	9,19	0.15
B	C	0.71	1	0.72	1	0.64	0.98	0.38	0.97	1.00	1	0.58	0.21	0.80	0.96	1	0.96	1.00	1	0.38
	D	1	0.84	0.98	1	0.03*	0.78	0.70	0.79	1	0.54	0.44	0.93	1	0.38	1	0.94	0.99	0.45	1
	E	1	1	0.71	0.99	0.19	0.74	0.22	1.00	0.97	0.76	0.11	1	0.96	1	0.21	0.97	1	0.97	1
C	D	0.63	0.80	0.34	1	0.39	0.97	0.98	0.098	1	0.72	0.99	0.59	0.87	0.13	1	0.99	1	0.75	0.25
	E	0.68	0.99	0.09	0.97	0.88	0.96	1	0.99	1	0.51	0.84	0.16	0.98	0.94	0.43	1	1	1	0.49
D	E	1	0.93	0.94	0.99	0.92	1	0.90	0.86	1	0.05	0.99	0.83	0.99	0.61	0.27	0.99	1	0.73	0.98

*p < 0.05.

Next, we conducted an individual data analysis (Table 12; one-way ANOVA, followed by the Tukey–Kramer *post-hoc* test). The results indicated that one participant exhibited a significantly reduced wakefulness duration in the early morning during and after violet light exposure, categorized as the symptomatic group. The effect was maintained for at least one month after exposure (participant 4). In addition, four participants tended to display a reduced wakefulness duration in the early morning during light exposure, although no or only partially significant differences were observed. Among them, one participant was categorized in the symptomatic group (participants 2, 5, 18, and 23). Within this group, violet light was most effective within two weeks after the start of light exposure in two participants, and the effect was attenuated within two weeks after the end of light exposure. In two of the other participants, the effect of light exposure lasted up to two weeks and four weeks after the end of exposure. In contrast, one participant showed a significant increase in the duration of wakefulness in the early morning during light exposure (participant 27). In summary, violet light exposure could potentially reduce the time spent in wakefulness in the early morning by approximately 26% in healthy persons, regardless of their symptoms. In contrast, approximately 5% of the healthy individuals might suffer a counterproductive effect.

From another perspective, early morning awakening can be defined as a wake-up time between 2:00 and 5:00 am in advanced sleep phase disorders [28]. However, in this study, two participants exhibited this condition only once during the control period; therefore, they did not meet the inclusion criteria.

### Violet light alters blood glucose levels relevant to sleep quality change

#### Susceptible and non-susceptible groups.

The effects of violet light on sleep experience are summarized in Table 13. Among the participants, 16% (indicated as ### in Table 13; participants 4, 16, and 17) showed a significant improvement in one or more categories of sleep experience and 37% (indicated as ## in Table 13; participants 2, 5, 18, 23, 24, 25, and 26) showed an improvement but without statistical significance. Combined, nearly half the participants showed some potential improvement in the overall sleep experience (referred to as the susceptible group; the others are referred to as the non-susceptible group). Among the participants in the susceptible group, 70% reported subjective improvement. Furthermore, in this group, 80% showed an improvement trend until one month after the completion of violet light exposure, and 20% showed improvement until two weeks after the end of exposure. On the contrary, 11% of all participants experienced counterproductive effects in one or more categories (++ in Table 13, referred to as the side effect group; paticipants 13 and 27). However, they all subjectively felt improvement, suggesting potential issues with data analysis, the accuracy of the acquired data, and/or bias in the number of data collections. Furthermore, 32% of participants did not show objective improvement but subjectively reported improvement (+ in Table 13, referred to as the placebo group; participants 8, 14, 19, 21, 26, and 29).

**Table 13:** Summary of violet light effect on sleep experience.

Objective effect		Subjective effect	
	Significance	(+)	(-)
(+)	(+)	1^###^	2^###^
	(-)	6^##^	1^##^
(+/-)		6^+^	2
(-)		2^++^	0
		
### and ##		Susceptible group	
+		Placebo group	
++		side effect group	

This result suggests a possible discrepancy between the subjective and objective effects of Violet light. Therefore, we analyzed and compared the biometric data between the susceptible and non-susceptible groups.

#### Verbal questionnaire results.

Previous studies have indicated that the quality of NREM sleep is associated with glucose homeostasis [17], and that violet light might influence blood glucose levels through its effects on the hypothalamus. Therefore, we used an verbal questionnaire to investigate changes in appetite and bowel movements and their relationship with violet light exposure and blood glucose levels.

**Fig 3:**
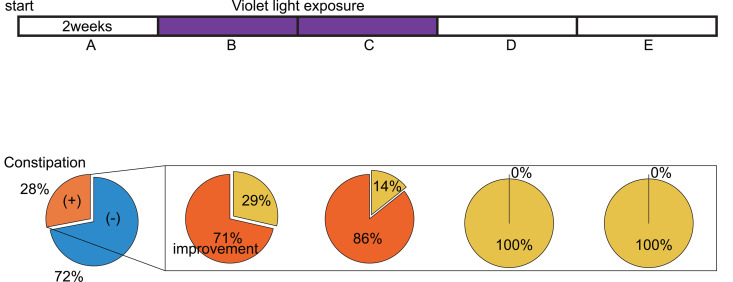
Time course changes in the bowel movement of the participants upon violet light exposure. The left column shows the rate of constipation during the control period, and the other column shows improvement during and after violet light exposure. Time periods A–E correspond to Fig 1.

The results showed that 40% (10 out of 25 participants) reported an increase in appetite after four weeks of violet light exposure, whereas no one complained of a decreased appetite. Among them, the appetite of 60% (6 individuals) returned to their baseline within four weeks after the end of violet light exposure, while 40% (4 individuals) experienced a sustained increase in appetite. As for the bowel movements, just under 30% of participants originally had constipation (Fig 3). Among them, nearly 90% experienced an improvement during violet light exposure, and this effect ceased after light exposure ended.

Next, we performed continuous blood gulcose level monitoring at each time point using the FreeStyle Library system. The following analyses were performed on 17 of the 19 participants for whom sleep data were analyzed, excluding two participants whose blood glucose data were incomplete.

#### Blood glucose levels throughout the day.

**Fig 4:**
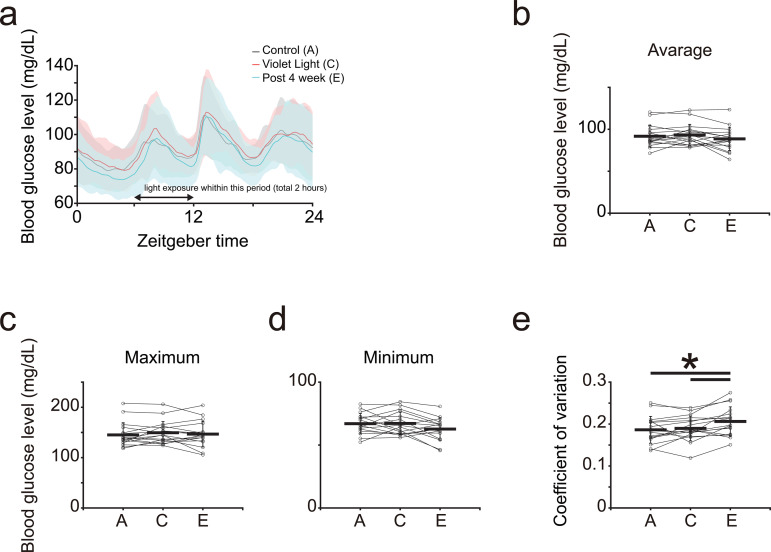
Blood glucose level alterations upon violet light exposure. **(a)** Daily variation in blood glucose (BS) levels. The black line shows the BS levels of the control period, the red line shows that of the period 2 to 4 weeks after the start of violet light exposure, and the cyan line shows that of 2 to 4 weeks after the end of violet light exposure. **(b)** Daily average of blood glucose levels. **(c)** Maximum blood glucose levels in a day. **(d)** Minimum blood glucose levels in a day. **(e)** Coefficient of variation in whole-day blood glucose levels. Each dot shows the mean of each participant. A, C, and E correspond to Figs. 1 and 3. Color shading and error bars indicate the SD. *p < 0.05.

We analyzed the blood glucose levels of each participant throughout the day and observed significant changes during and after violet light exposure. The average blood glucose (BS) levels throughout the day indicated peak BS levels in the morning during violet light exposure; the mean and minimum BS levels decreased after violet light exposure (Fig 4a).

Subsequently, we compared the average, maximum, and minimum BS levels throughout the day and divided the participants into two groups. Regarding average values, one group showed changes in BS levels only during violet light exposure (participants 2, 4, 16, 19, 23, 24, and 26), while the other group showed changes during and after violet light exposure (participants 5, 15, 20, 26, and 29; Figs 4b–4d, Table 14), indicating no consistent trend. Among all participants, 17.6% exhibited significantly increased BS levels (average and either or both maximum or minimum value) during light exposure compared to before and after exposure (participants 16, 23, and 24); no one showed an increasing trend (Table 15; repeated measures ANOVA, followed by the Tukey–Kramer *post-hoc* test). Meanwhile, 5.9% of participants showed significantly decreased BS levels during light exposure compared to before and after exposure (participant number: 19), and 17.6% of participants showed a decreasing trend (participantss 2, 4, and 26; Table 15; repeated measures ANOVA, followed by the Tukey–Kramer *post-hoc* test). All participants in the “increased” group (participants 16, 23, and 24) and half of those in the “decreased” group (participants 2 and 4) showed an improvement in sleep quality; the effect of violet light on sleep quality changes and BS level changes are independent (**p* *= 0.15 in Pearson’s chi-square test).

**Table 14:** Average, maximum, and minimum blood glucose levels throughout the day in each participant.

Subject number	Blood glucose level (mg/dL)								
	Time period								
	A			C			E		
	Av.	Max.	Min.	Av.	Max.	Min.	Av.	Max.	Min.
2	95.6 ± 2.7	134.3 ± 8.8	69.8 ± 7.2	85.1 ± 10.9	126.5 ± 17.1	60.6 ± 7.5	94.4 ± 11.9	145.7 ± 12.6	66.5 ± 9.9
4	85.5 ± 7.3	139.1 ± 22.5	62.5 ± 7.5	79.5 ± 11.8	134.2 ± 24.0	57.8 ± 11.3	87.7 ± 5.4	142.3 ± 20.3	63.1 ± 10.9
5	117.1 ± 8.7	191.1 ± 21.1	76.2 ± 6.8	122.9 ± 4.7	188.2 ± 7.0	84.4 ± 5.4	123.5 ± 12.0	203.9 ± 24.3	80.7 ± 11.5
8	86.8 ± 5.6	125.4 ± 14.3	65.5 ± 9.1	89.8 ± 7.0	124.1 ± 17.5	70.3 ± 6.2	72.6 ± 15.9	105.5 ± 20.9	54.7 ± 10.9
13	91.3 ± 2.6	135.8 ± 17.0	72.6 ± 4.1	93.6 ± 3.1	144.3 ± 22.4	61.7 ± 7.1	78.7 ± 8.6	131.7 ± 27.6	45.7 ± 9.8
14	120.4 ± 6.3	207.6 ± 24.5	82.5 ± 7.2	118.2 ± 5.9	206.0 ± 24.1	81.8 ± 4.7	105.6 ± 3.9	184.4 ± 19.6	72.3 ± 5.1
15	104.1 ± 5.0	160.2 ± 18.3	79.4 ± 7.1	96.0 ± 10.3	148.5 ± 18.8	65.8 ± 11.9	96.6 ± 3.2	151.1 ± 11.3	70.9 ± 13.3
16	84.8 ± 2.7	129.8 ± 8.6	64.0 ± 3.8	99.7 ± 7.3	162.2 ± 16.3	76.9 ± 9.2	90.5 ± 3.6	148.7 ± 16.9	66.5 ± 5.2
17	96.1 ± 4.4	147.8 ± 13.4	72.2 ± 4.3	98.2 ± 5.4	152.3 ± 15.5	72.8 ± 6.7	90.8 ± 8.4	145.5 ± 13.3	66.4 ± 7.9
19	89.5 ± 5.3	137.2 ± 14.3	65.5 ± 11.7	81.2 ± 3.8	127.7 ± 15.1	58.5 ± 5.8	86.2 ± 2.9	139.9 ± 16.3	63.1 ± 7.2
20	85.8 ± 6.1	149.2 ± 20.6	63.4 ± 6.7	91.9 ± 0.9	165.8 ± 20.8	65.6 ± 1.8	89.0 ± 3.8	176.6 ± 14.3	61.1 ± 8.4
21	80.7 ± 6.8	130.2 ± 18.0	61.0 ± 8.3	88.5 ± 4.1	144.8 ± 11.4	59.2 ± 9.7	64.1 ± 15.2	108.5 ± 24.5	46.4 ± 8.9
23	71.4 ± 12.4	118.3 ± 21.0	52.3 ± 10.3	86.9 ± 4.7	135.9 ± 9.8	64.3 ± 10.4	71.5 ± 8.4	120.5 ± 15.0	54.1 ± 8.5
24	88.0 ± 3.2	135.3 ± 21.5	66.5 ± 5.1	92.4 ± 2.5	153.0 ± 14.3	67.8 ± 9.1	88.3 ± 5.8	148.8 ± 13.4	65.5 ± 6.2
25	78.1 ± 3.3	120.6 ± 17.0	55.3 ± 8.5	78.2 ± 4.9	131.6 ± 16.3	55.9 ± 6.7	85.6 ± 1.9	133.5 ± 18.9	61.3 ± 8.5
26	99.8 ± 4.4	165.7 ± 18.8	70.8 ± 10.4	103.2 ± 2.6	158.5 ± 19.6	78.5 ± 5.3	99.9 ± 3.6	168.5 ± 20.5	68.3 ± 11.6
29	84.1 ± 5.7	140.2 ± 17.9	59.1 ± 9.8	81.4 ± 3.5	136.5 ± 15.7	59.4 ± 6.9	81.0 ± 5.3	139.3 ± 19.4	59.5 ± 4.5

**Table 15:** Statistical analysis results of the average blood glucose levels throughout the day in each participant.

Comparison		*p* Value																
		Subject number																
		2	4	5	8	13	14	15	16	17	19	20	21	23	24	25	26	29
A	C	0.02*	0.18	0.47	0.75	0.63	0.59	1.5 × 10^−2^*	1.3 × 10^−8^*	0.69	2.7 × 10^−5^*	0.06	0.12	3.1 × 10^−4^*	0.02*	1	0.06	0.34
	E	0.95	0.80	0.32	4.1 × 10^−3^*	3.5 × 10^−4^*	6.4 × 10^−7^*	0.04*	0.01*	0.10	0.11	0.29	3.8 × 10^−4^*	1	0.98	5.1 × 10^−5^*	1	0.26
C	E	0.05	5.0 × 10^−2^*	0.99	5.3 × 10^−4^*	7.2 × 10^−3^*	3.5 × 10^−6^*	0.97	1.0 × 10^−4^*	0.02*	9.9 × 10^−3^*	0.54	8.2 × 10^−7^*	3.3 × 10^−4^*	0.04*	5.3 × 10^−5^*	0.07	0.98

*p < 0.05.

However, some participants showed changes in their BS levels only after light exposure (participants 8, 13, 14, 17, 21, and 25). Among all participants, 29.4% showed significantly decreased BS levels only after the end of exposure compared to the control period (participants 8, 13, 14, 17, and 21), while 11.8% of participants exhibited significantly increased BS levels only after the end of exposure (participant 25; Tables 14 and 15). This result was significantly relevant to sleep quality change (*p* = 0.01 in Pearson’s chi-square test); that is, improved sleep quality is related to a decrease in blood glucose levels after violet light exposure, and vice versa.

Furthermore, the coefficient of variation (CV) of the BS levels throughout the day significantly increased after violet light exposure (Fig 4e). However, individual data analysis showed that only 18% of participants showed significantly increased CVs in BS levels (participants 2, 13, and 20; Table 16; repeated measures ANOVA, followed by the Tukey–Kramer *post-hoc* test), suggesting that the individual CV alterations might be slight but showed an increasing trend.

**Table 16:** Statistical analysis results of the coefficient of variation of blood glucose levels throughout the day in each participant..

Comparison		*p* Value																
		Subject number																
		2	4	5	8	13	14	15	16	17	19	20	21	23	24	25	26	29
A	C	0.04*	0.88	0.68	0.44	0.34	0.96	0.49	0.32	0.61	0.98	0.86	0.74	0.86	0.65	0.69	0.05	0.97
	E	2.7 × 10^−3^*	0.93	0.92	0.84	6.1 × 10^−3^*	0.85	0.96	0.20	0.91	0.89	6.1 × 10^−3^*	0.16	0.93	0.18	0.98	0.89	0.79
C	E	0.51	0.99	0.55	0.18	0.24	0.65	0.69	0.96	0.85	0.80	0.10	0.50	0.66	0.63	0.60	0.14	0.90

*p < 0.05.

#### Blood glucose levels in each time period.

**Fig 5:**
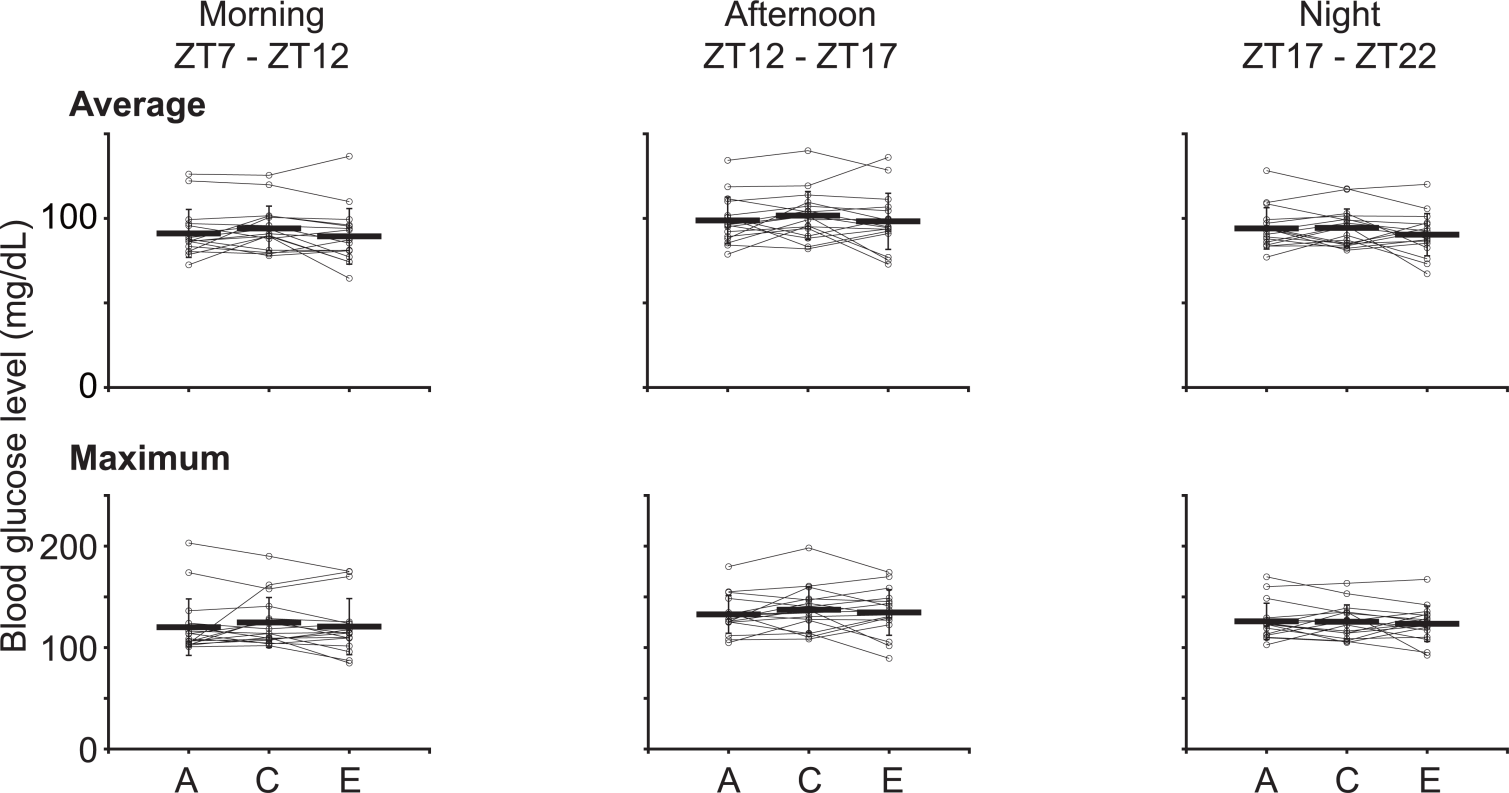
Daily variation in the average and maximum blood glucose (BS) level upon violet light exposure. Average and peak BS levels during each time period of the day. The left column shows the BS levels in the morning (ZT7 to ZT12), the middle column shows those in the afternoon (ZT12 to ZT17), and the right column shows those at night (ZT17 to ZT22). The upper row shows the average BS levels and the lower row shows the peak BS levels.

As discussed in the previous section, violet light-induced BS changes occur during and after light exposure. Therefore, we considered that the postprandial BS level might be changed by violet light exposure and divided each day into three segments: 7:00 am to 12:00 pm, which roughly overlaps with the period of violet light exposure and breakfast time; 12:00 pm to 5:00 pm, which roughly overlaps with lunch and snack time; and 5:00 pm to 10:00 pm, which roughly overlaps with dinner time. We then examined the average and individual BS levels during each time interval (Fig 5). The average BS levels in the first period were 91.1 ± 14.2, 94.1 ± 13.2, and 89.4 ± 16.4 mg/dL (time periods A, C, and E, respectively), those in second period were 98.8 ± 13.8, 101.6 ± 14.2, 98.3 ± 16.6 mg/dL (time periods A, C, and E, respectively), and those in third period were 94.1 ± 12.2, 94.4 ± 11.2, 90.4 ± 12.5 mg/dL (time periods A, C, and E, respectively). These data collectively show that changes in BS levels do not occur at specific time intervals of the day (morning, noon, or evening), but rather occur throughout the entire day (Table 17; Repeated measures ANOVA, followed by the Tukey–Kramer *post-hoc* test). This trend was observed in the maximum BS level (Table 17).

**Table 17:** Statistical analysis results of the daily average and maximum blood glucose level variations.

Comparison		*p* Value					
		Morning		Afternoon		Night	
		Average	Maximum	Average	Maximum	Average	Maximum
A	C	0.35	0.54	0.48	0.39	0.99	0.99
	E	0.72	0.99	0.97	0.78	0.27	0.73
C	E	0.14	0.37	0.49	0.79	0.34	0.87

*p** < 0.05.**

### Violet light might improve the eye environment

**Fig 6:**
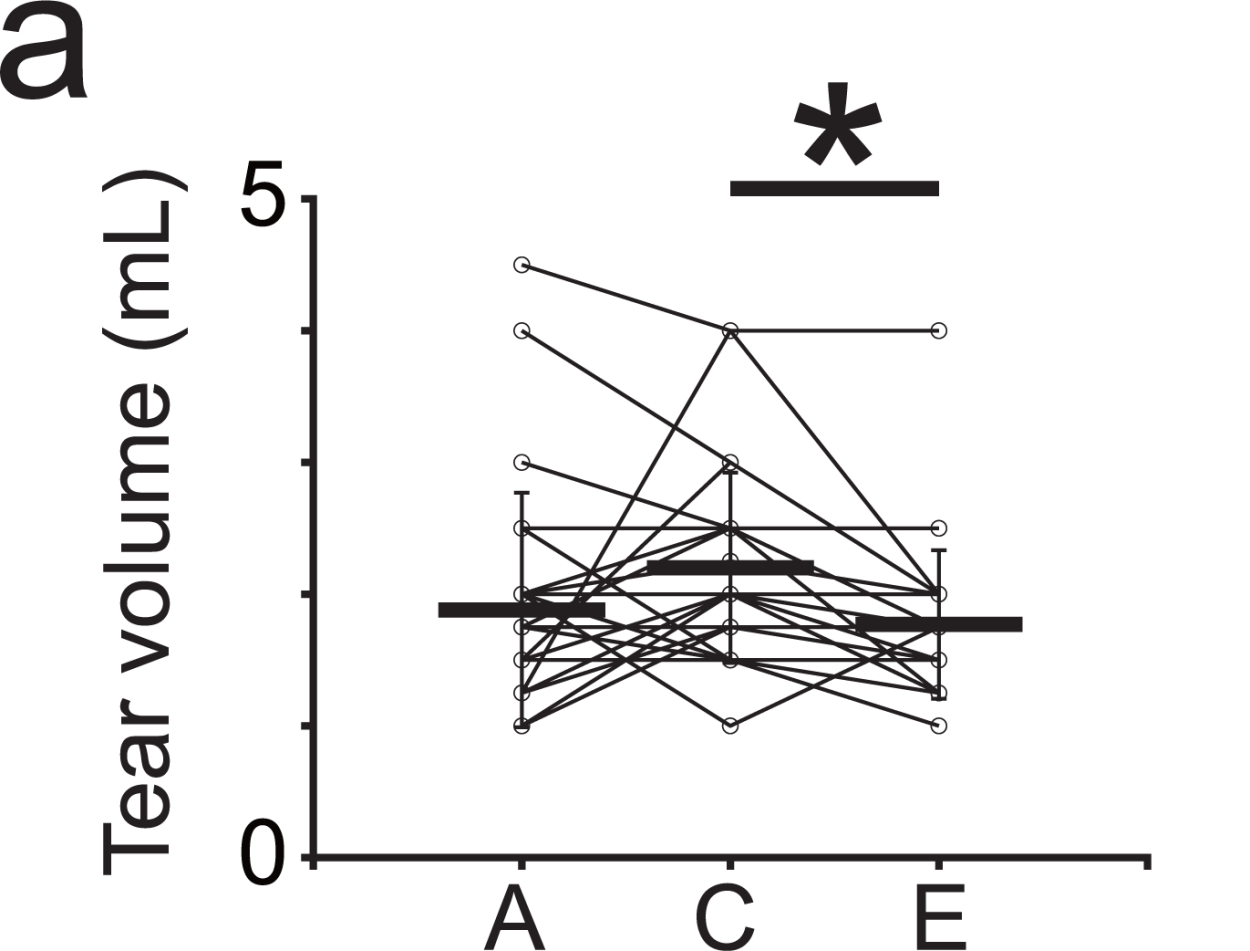
Mean tear volume upon violet light exposure. Mean tear volume before **(A)**, during **(C)**, and after (E) violet light exposure.

Next, we analyzed the tear volume to determine whether violet light exposure affected the eye environment. The average tear volume in both eyes before violet light exposure was 1.88 ± 0.89 mL (mean ± SD). It increased to 2.20 ± 0.72 mL after four weeks of violet light exposure, but it was not statistically significant (Fig 6A and 6C; *p* = 0.17, Repeated measures ANOVA, followed by the Tukey–Kramer *post-hoc* test). After that, four weeks after violet light exposure, the tear volume significantly decreased to 1.77 ± 0.56 mL (Fig 6C and 6E; *p* = 1.6 × 10^−3^, Repeated measures ANOVA, followed by the Tukey–Kramer *post-hoc* test), returning to the baseline (Fig 6A and 6E; *p* = 0.70, Repeated measures ANOVA, followed by the Tukey–Kramer *post-hoc* test). These results suggest that there is a tendency for the tear volume to increase during violet light exposure (Table 18; The sequence is independent of the participant numbers used in other datasets and was encrypted using a reversible method to allow for potential re-identification if necessary).

**Table 18:** Tear volume results before, during and after violet light exposure.

		*p* Value			
Comparison		ZT 2–7	ZT 7–12	ZT 12–17	ZT 17–22
pre	Light on 2 wk	0.99	0.96	0.96	1
	Light on 4 wk	0.97	0.99	0.76	1
	Light off 2 wk	0.99	0.84	0.76	0.98
	Light off 4 wk	0.99	0.94	0.97	0.98

*p < 0.05.

**wk: week.**

### Violet light exposure does not affect the heart rate

Finally, we investigated the changes in heart rate during the experiment upon violet light exposure. OPN5, the receptor for sensing violet light, is expressed in QRFP (alias name QPLOT) neurons in the preoptic area (POA) of the hypothalamus in mice [12,29,30], and stimulation of these neurons decreases the heart rate [31]. Therefore, we expected changes in the heart rate if violet light directly stimulated the hypothalamus via OPN5 receptors.

Our results showed that the mean heart rate during and after violet light exposure was not significantly different from that before violet light exposure (Fig 6a, Table 19; repeated measures ANOVA followed by the Tukey–Kramer *post-hoc* test).

**Table 19:** Statistical analysis results of the mean heart rates in each period before, during, and after violet light exposure.

midnight						
			*p* Value			
Comparison		Subject Number	ZT 2–7	ZT 7–12	ZT 12–17	ZT 17–22
pre	Light on 2 wk	2	0.83	0.36	0.35	0.46
		4	0.98	0.44	0.08	0.62
		5	0.20	0.11	2.5 × 10^−4^*	0.02*
		8	0.44	0.06	0.07	2.4 × 10^−3^*
		13	7.9 × 10^−4^*	0.78	0.07	0.50
		15	0.12	9.2 × 10^−3^*	0.63	0.85
		16	0.42	0.15	0.97	0.34
		18	0.03*	0.08	0.25	4.8 × 10^−2^*
		19	0.04*	0.06	0.99	0.47
		20	0.60	0.44	0.27	0.07
		21	2.3 × 10^−3^*	0.22	0.57	0.40
		23	0.18	0.94	0.85	4.9 × 10^−2^*
		24	2.1 × 10^−4^*	6.4 × 10^−3^*	0.01*	0.02*
		26	0.33	0.36	0.01*	0.58
		27	0.18	0.15	0.72	0.17
		29	0.83	0.79	0.49	0.16
morning						
			*p* Value			
Comparison		Subject Number	ZT 2–7	ZT 7–12	ZT 12–17	ZT 17–22
pre	Light on 4 wk	2	0.35	0.58	0.24	0.83
		4	0.88	0.07	0.80	0.32
		5	0.21	0.22	0.09	0.29
		8	0.56	1	0.78	2.5 × 10^−3^*
		13	0.48	0.78	0.09	0.82
		15	0.73	0.78	0.48	0.71
		16	0.01*	1.2 × 10^−3^*	5.0 × 10^−3^*	4.9 × 10^−5^*
		18	0.82	0.80	0.30	0.78
		19	0.12	0.02*	0.92	0.44
		20	0.08	0.04*	0.10	0.15
		21	9.0 × 10^−3^*	0.85	0.38	0.15
		23	0.19	0.88	0.93	0.87
		24	0.20	0.38	0.49	0.14
		26	0.30	0.03*	0.01*	0.13
		27	0.95	2.1 × 10^−5^*	1.1 × 10^−3^*	0.03*
		29	0.38	0.36	0.10	0.03*
Afternoon						
			*p* Value			
Comparison		Subject Number	ZT 2–7	ZT 7–12	ZT 12–17	ZT 17–22
pre	Light off 2 wk	2	0.58	0.41	0.27	0.43
		4	0.40	0.18	0.95	0.40
		5	0.65	0.88	0.35	0.85
		8	0.53	0.80	0.90	8.1 × 10^−3^*
		13	0.03*	0.88	0.78	0.25
		15	0.66	0.28	0.70	0.95
		16	0.34	0.06	0.27	0.03*
		18	0.55	0.45	0.72	0.33
		19	0.47	0.28	0.65	0.34
		20	0.69	0.72	0.38	0.76
		21	0.92	4.4 × 10^−3^*	0.13	0.13
		23	0.19	0.66	0.90	0.62
		24	0.13	0.10	0.20	0.31
		26	0.60	0.93	0.35	0.42
		27	0.024	5.1 × 10^−5^*	0.02*	0.01*
		29	0.01*	0.30	0.21	0.02*
Night						
			*p* Value			
Comparison		Subject Number	ZT 2–7	ZT 7–12	ZT 12–17	ZT 17–22
pre	Light off 4 wk	2	0.66	0.83	0.10	0.74
		4	0.86	0.53	0.36	0.61
		5	0.12	0.26	0.03*	0.34
		8	0.20	0.38	0.26	4.3 × 10^−4^*
		13	0.02*	0.69	0.23	0.03*
		15	0.17	7.1 × 10^−3^*	0.15	0.01*
		16	0.30	0.04*	0.22	9.2 × 10^−3^*
		18	0.86	0.40	0.85	0.30
		19	0.90	0.53	1.5 × 10^−4^*	0.13
		20	0.43	0.17	0.81	0.92
		21	0.27	0.09	0.68	0.76
		23	0.27	0.82	0.30	0.98
		24	0.12	0.41	0.52	0.22
		26	0.92	0.60	0.35	0.52
		27	0.04*	9.3 × 10^−5^*	8.4 × 10^−3^*	3.7 × 10^−3^*
		29	4.8 × 10^−3^*	4.8 × 10^−2^*	9.7 × 10^−4^*	0.02*

*p < 0.05.

wk: week.

Next, we analyzed the data per individual (Table 20; linear mixed-effects model) and found that 13% of the participants showed a significant change (increase or decrease) in heart rate during the morning (ZT 7–12) up to the second week after light exposure, 31% up to the fourth week, 13% and 25% after two and four weeks post-exposure, respectively, showing no consistent pattern. Significant differences in heart rate in any of the time periods, i.e., midnight (ZT 2–7), morning (ZT 7–12), afternoon (ZT 12–17), or evening (ZT17–22), were observed in 63%, 50%, 38%, and 50% of participants, respectively, also showing no consistent trend.

**Table 20:** Statistical analysis results of the violet light exposure-related mean heart rates in each participant.

midnight					
	avarage heart rate				
Subject Number	pre	Light on 2 wk	Light on 4 wk	Light off 2 wk	Light off 4 wk
2	73.7	74.2	71.7	71.6	72.1
4	55.0	55.1	53.9	52.4	54.3
5	67.6	66.3	65.8	63.4	65.0
8	60.8	62.4	64.7	64.5	67.1
13	64.1	69.4	64.5	66.9	62.6
15	67.9	70.5	72.8	67.1	68.2
16	64.4	66.7	68.5	64.3	67.2
18	58.3	55.4	55.8	56.6	55.7
19	71.4	67.4	67.8	71.4	72.7
20	57.0	56.4	56.1	55.6	54.2
21	56.5	65.9	52.9	58.1	54.0
23	60.8	59.4	60.8	61.1	58.6
24	69.0	64.3	65.5	65.9	66.0
26	68.5	70.3	70.2	73.5	69.5
27	72.3	69.9	69.7	71.6	69.8
29	78.4	77.7	77.6	76.3	82.7
morning					
	avarage heart rate				
Subject Number	pre	Light on 2 wk	Light on 4 wk	Light off 2 wk	Light off 4 wk
2	90.5	92.5	91.7	93.1	91.0
4	75.5	76.1	69.1	76.5	72.2
5	85.7	81.8	81.8	80.4	82.4
8	83.7	81.3	83.7	84.8	96.1
13	87.5	88.7	87.9	90.7	86.8
15	82.7	83.5	82.0	81.1	81.8
16	74.9	82.9	85.3	83.9	89.0
18	80.9	79.8	79.6	86.6	79.6
19	91.3	87.6	85.7	91.6	89.5
20	74.3	79.7	80.7	80.0	80.2
21	75.1	84.6	74.4	78.1	80.1
23	81.5	78.5	81.8	81.3	81.9
24	86.8	83.7	84.6	85.1	83.2
26	80.2	82.3	84.8	85.6	83.3
27	91.4	91.5	82.3	84.6	86.9
29	91.8	89.9	90.0	88.3	87.0
Afternoon					
	avarage heart rate				
Subject Number	pre	Light on 2 wk	Light on 4 wk	Light off 2 wk	Light off 4 wk
2	93.0	94.4	95.0	95.8	95.0
4	80.5	77.2	75.1	80.3	77.3
5	82.7	81.5	82.3	80.3	82.2
8	86.3	83.6	85.2	86.8	97.4
13	87.6	91.4	87.3	88.1	84.6
15	79.4	80.7	82.9	78.2	79.2
16	80.5	83.3	86.1	83.7	87.3
18	83.6	86.0	86.7	85.2	79.9
19	93.0	90.9	89.7	91.6	90.2
20	82.3	84.1	83.9	78.0	80.5
21	83.3	83.6	73.8	78.3	78.3
23	81.1	78.5	80.3	80.9	80.2
24	83.7	80.7	80.3	81.1	81.7
26	80.7	82.5	80.4	84.0	83.4
27	97.0	94.1	86.1	91.1	90.6
29	91.5	87.0	89.9	89.4	87.4
Night					
	avarage heart rate				
Subject Number	pre	Light on 2 wk	Light on 4 wk	Light off 2 wk	Light off 4 wk
2	92.2	93.7	92.9	97.9	91.0
4	75.5	75.0	74.0	73.3	74.3
5	85.5	82.2	83.0	80.6	83.4
8	80.2	85.3	83.6	84.5	94.1
13	82.7	86.3	82.1	84.5	77.9
15	75.3	79.4	84.2	79.5	82.9
16	78.2	81.4	84.8	82.0	86.8
18	77.1	76.7	75.1	76.7	79.4
19	84.4	84.6	83.5	90.0	86.4
20	73.5	76.0	77.9	74.3	73.1
21	79.0	81.5	75.2	79.9	78.3
23	77.1	75.5	77.4	78.6	77.0
24	80.6	78.3	79.3	79.6	78.7
26	81.6	81.9	82.9	84.0	83.2
27	91.2	86.5	81.6	84.9	84.3
29	91.0	84.6	87.0	83.4	85.7

*p < 0.05

wk: week.

Finally, we analyzed the trends in the average heart rate regardless of the statistical significance; however, no consistent change was observed during the exposure period (morning period; Table 21). However, 18.8% of participants (participants 4, 8, and 24) showed decreased heart rates during the afternoon period (Table 20), while 18.8% (participants 15, 18, and 20) showed increased heart rates (Table 21).

**Table 21:** Mean heart rate related to violet light exposure in each participant.

Tear volume (mL)		
Time period		
A	C	E
2	2	1.75
1.75	2.5	1.25
2	2.5	1.75
1	2	1.25
1.25	4	2
1.5	1.5	1.25
2.5	1.5	1
2	1	1.75
3	2.5	2.5
1	2	1.5
1.25	2	1.5
1	2	2
1.75	1.5	1.5
1.5	2	1.75
2.5	2.5	1.75
1	2	1.5
4.5	4	4
1	1.75	1.75
1.75	1.75	1.5
2	2.25	2
4	3	2
1.5	3	2
2	2.5	1.75
1.25	1.75	1.75
2	1.5	1.5

## Discussion

Our study revealed that violet light could subjectively enhance sleep quality and bowel movements. Our objective data suggest that violet light exposure might significantly improve sleep quality in approximately 20–30% of the healthy individuals. Moreover, violet light exposure potentially influenced BS levels, with 20% of participants experiencing an increase and a similar percentage a decrease. In addition, tear volume tended to increase upon violet light exposure, hinting potential eye protection. Taken together, our results indicated broad physiological impact upon violet light exposure.

One mechanism that controls the sleep-wake cycle is the circadian rhythm, which establishes a period of approximately 24 h [32], the suprachiasmatic nucleus (SCN) being its pacemaker, controlled by light. The retina, composed of retinal ganglion cells, receives light, and this photic information is transmitted to the SCN via the retinohypothalamic tract [33]. SCN neurons reportedly adjust their circadian phase [34]. Therefore, natural daylight during the day improves sleep quality [35], and artificial light exposure could exert therapeutic effects on circadian rhythm disorders [36]. Although OPN5, a violet light receptor, is distributed in the retina, *OPN5*-expressing retinal ganglion cells (*OPN5*-RGCs) displpay limited expression and a specific projection pattern that does not project to the SCN [37]. Previous studies described that *OPN5*-RGCs project to the supraoptic nucleus [37] and connect to *OPN5*-positive POA neurons, which overlap with QRFP neurons [12,30]. QRFP neurons are primarily involved in body temperature control [31]. However, whether these neurons control the sleep-wake cycle remains unclear. Body temperature alterations can reportedly affect sleep-wake regulation in a delayed manner [38–40]. Therefore, body temperature measurements during the experiment and at night would be required to determine how violet light improves sleep quality. However, the relationship between artificial daytime temperature variations and nighttime sleep quality remains unclear. However, in this study, temperature changes induced in the participants during the morning might have potentially affected sleep quality later at night.

Previous studies indicated that OPN5 is expressed in the preoptic area (POA) of the brain [12,30]. As the POA is involved in sleep-wake cycle, body temperature, and heart rate regulation [31,41–43]. Therefore, direct POA stimulation with violet light might produce potent biological effects. However, in humans, the POA is located in the deep brain area. Moreover, as visible light only penetrates the skin up to the reticular dermis [44], violet light expectably cannot reach the deep brain area. In our study, violet light did not alter the heart rate, which potentially also supports this hypothesis.

Furthermore, our results revealed that blood glucose level improvements during violet light exposure was independent of sleep quality improvements. However, after violet light exposure, blood glucose level and sleep quality improvements were mutually related. As appropriate NREM sleep reportedly induces superior blood glucose control the upcoming day [17,45] and insomnia could increase the risk of diabetes [18,46], blood glucose level improvements upon violet light exposure is likely a secondary effect resulting from improved sleep. In other words, violet light might not directly affect blood glucose levels. However, since light is also known to affect mood [4], the stabilization of which being closely linked to that of eating behavior [47], violet light exposure-induced blood glucose level fluctuations would not be surprising.

The limitations of this study include its small sample size and the need for improvements in the experimental methods used during the control period. Concerning the sample size, experiments with larger sample sizes will be necessary in the future. Regarding the experimental methods, due to the discrepancies between the subjective and objective results, placebo effect likely influenced significantly the results of this study. Considering this, gathering data over a longer period and conducting a parallel study using “placebo” glasses that emit a different color of light but are identical to the violet light-emitting glasses might have been a better approach. In future studies, the use of a placebo-control light, such as white light or colored light with no known physiological effects, is crucial to clearly the unique effects of violet light. In addition, our data cannot determine whether sleep quality improvement results from violet light exposure or is simply due to normal sleep fluctuations, so that it might require a negative control group, which only records biometric data for the same periods. The results of this study suggest that when the area around the eyes is irradiated with violet light, sleep improves compared to no irradiation (i.e., exposure to natural sunlight), and secondary effects are associated with this. However, whether such OPN5-mediated effects mediated are stronger or weaker than those mediated by conventional photoreceptors cannot be determined without irradiating the area around the eye with white light of the same intensity. Certainly, considering the potential commercialization of these glasses, violet light represents an additional value, e.g., its effect in suppressing myopia [9], there would thus likely be a demand even without these data. However, demonstrating that violet light offers more benefits than white light increases its promotional impact. Finally, the accuracy of the Fitbit device used to assess sleep quality in this study was approximately 60% [27], potentially affecting the discrepancy between subjective and objective results. Although it is commonly used in human studies, future research should use more accurate devices for validation.

## Conclusions

Our results indicated that violet light irradiation might contribute to regulating blood glucose levels and improving sleep onset in certain individuals, thereby suggesting the potential application of violet light in sleep and metabolic health management, particularly in individuals with delayed sleep onset or increased blood glucose levels. Overall, although the results of this study do not suggest that violet light is a miracle cure, it might offer other benefits, such as when used as a health supplement.
